# Glial activation and inflammation along the Alzheimer’s disease continuum

**DOI:** 10.1186/s12974-019-1399-2

**Published:** 2019-02-21

**Authors:** Kaja Nordengen, Bjørn-Eivind Kirsebom, Kristi Henjum, Per Selnes, Berglind Gísladóttir, Marianne Wettergreen, Silje Bøen Torsetnes, Gøril Rolfseng Grøntvedt, Knut K. Waterloo, Dag Aarsland, Lars N. G. Nilsson, Tormod Fladby

**Affiliations:** 1 Department of Neurology, Akershus University Hospital, P.B. 1000, N-1478 Lørenskog, Norway; 2 Department of Neurology, University Hospital of North Norway, Tromsø, Norway; 3 Department of Psychology, Faculty of Health Sciences, UiT The Arctic University of Norway, Tromsø, Norway; 4 Department of Pharmacology, Institute of Clinical Medicine, University of Oslo and Oslo University Hospital, Oslo, Norway; 5 Clinical Molecular Biology (EpiGen), Medical Division, Akershus University Hospital and University of Oslo, Oslo, Norway; 6 Department of Neurology and Clinical Neurophysiology, University Hospital of Trondheim, Trondheim, Norway; 7 Centre for Age-Related Medicine, Stavanger University Hospital, Stavanger, Norway; 8 Department of Old Age Psychiatry, Institute of Psychiatry, Psychology and Neuroscience, King’s College London, London, UK; 9 Institute of Clinical Medicine, Campus Ahus, University of Oslo, Oslo, Norway

**Keywords:** Early diagnosis, Cerebrospinal fluid, ELISA, sTREM2, YKL-40, Chitinase-3-like protein 1, MCP-1, Monocyte chemoattractant protein-1, Fractalkine, CX3CL1, Clusterin, Apolipoprotein J, Microglia, Neuroinflammation

## Abstract

**Background:**

Neuronal and glial cell interaction is essential for synaptic homeostasis and may be affected in Alzheimer’s disease (AD). We measured cerebrospinal fluid (CSF) neuronal and glia markers along the AD continuum, to reveal putative protective or harmful stage-dependent patterns of activation.

**Methods:**

We included healthy controls (*n* = 36) and Aβ-positive (Aβ+) cases (as defined by pathological CSF amyloid beta 1-42 (Aβ42)) with either subjective cognitive decline (SCD, *n* = 19), mild cognitive impairment (MCI, *n* = 39), or AD dementia (*n* = 27). The following CSF markers were measured: a microglial activation marker—soluble triggering receptor expressed on myeloid cells 2 (sTREM2), a marker of microglial inflammatory reaction—monocyte chemoattractant protein-1 (MCP-1), two astroglial activation markers—chitinase-3-like protein 1 (YKL-40) and clusterin, a neuron-microglia communication marker—fractalkine, and the CSF AD biomarkers (Aβ42, phosphorylated tau (P-tau), total tau (T-tau)). Using ANOVA with planned comparisons, or Kruskal-Wallis tests with Dunn’s pairwise comparisons, CSF levels were compared between clinical groups and between stages of biomarker severity using CSF biomarkers for classification based on amyloid pathology (A), tau pathology (T), and neurodegeneration (N) giving rise to the A/T/N score.

**Results:**

Compared to healthy controls, sTREM2 was increased in SCD (*p* < .01), MCI (*p* < .05), and AD dementia cases (*p* < .001) and increased in AD dementia compared to MCI cases (*p* < .05). MCP-1 was increased in MCI (*p* < .05) and AD dementia compared to both healthy controls (*p* < .001) and SCD cases (*p* < .01). YKL-40 was increased in dementia compared to healthy controls (*p* < .01) and MCI (*p* < .05). All of the CSF activation markers were increased in subjects with pathological CSF T-tau (A+T−N+ and A+T+N+), compared to subjects without neurodegeneration (A−T−N− and A+T−N−).

**Discussion:**

Microglial activation as indicated by increased sTREM2 is present already at the preclinical SCD stage; increased MCP-1 and astroglial activation markers (YKL-40 and clusterin) were noted only at the MCI and AD dementia stages, respectively, and in Aβ+ cases (A+) with pathological T-tau (N+). Possible different effects of early and later glial activation need to be explored.

**Electronic supplementary material:**

The online version of this article (10.1186/s12974-019-1399-2) contains supplementary material, which is available to authorized users.

## Background

Alzheimer’s disease (AD) may be described as a biological continuum that includes the hallmark pathological processes of amyloid-beta (Aβ) dysmetabolism, formation of amyloid deposits (A), neurofibrillary tangles (T), neurodegeneration (N), determined by measuring cerebrospinal fluid (CSF) levels of Aβ42, phosphorylated tau (P-tau), and total tau (T-tau) respectively. The presence or absence of pathological markers can be summarized as an A/T/N score, an unbiased classification of pathology and severity along the AD continuum [1, 2]. In contrast, the clinical classification of the AD continuum is based on subjective accounts of cognitive deficits, performance on cognitive tests, and functioning in daily life [3–6]. Patients who report experience of decline in cognitive function while performing within the normal range on cognitive tests, may be categorized as having subjective cognitive decline (SCD) [3]. In contrast, mild cognitive impairment (MCI) requires the presence of subjective cognitive decline in combination with impaired cognitive performance yet retaining preserved independence in functional ability [4–6]. We and others have made large efforts towards standardization of criteria for these stages, e.g., as part of the EU JPND-funded BIOMARKAPD study, and Norwegian national efforts [7, 8].

While genetic evidence indicates that Aβ dysmetabolism is causal in familial AD, the initial sequence of events and causality in sporadic AD is still not determined. However, reduced Aβ clearance and deficient innate immune activity related to the triggering receptor expressed on myeloid cells 2 (TREM2) and clusterin (Apo J) function may play a role [9–11]. While central nervous system (CNS) interstitial Aβ is released from neurons dependent on activity, clearance is a result of neuronal, astro-, and microglial uptake and degradation as well as transport to the glymphatic system, blood, and (CSF) [12–14]. Microglia normally subserve synaptic homeostasis and synapse elimination [15, 16]. They are CNS myeloid-derived innate immune effector cells, which together with reactive astrocytes also may acquire inflammatory properties. Genetic evidence supports a role for loss of balanced TREM2 activation, innate immunity, and microglial activity in AD pathogenesis [9, 17–19]. Further, experimental studies support neuroinflammatory responses as drivers of AD pathogenesis, and there is evidence for associations to neuroinflammation and deficient microglia Aβ function in MCI due to AD and more advanced AD [14, 20–22], though the initial microglial activation might be compensatory and advantageous. Aβ clearance decreases with age and could in combination with genetic liabilities for compromised innate immune clearance capacity contribute to age-related disease inception [23, 24]. Notably, a recent translocator protein (TSPO) ligand positron-emission tomography (PET) study detecting activated microglia showed higher binding in AD “slow decliners” [25]. Moreover, a longitudinal TSPO-PET study demonstrated reduced microglia activation over time in patients at the MCI stage, but increased activation in patients at the AD stage of dementia [26]. These findings may be interpreted as an early beneficial role of microglial activation and a later inflammatory peak. Experimental evidence suggests that TREM2 increases in parallel with amyloid deposition, possibly limiting Aβ plaque-associated pathology [27, 28]. Thus, initial microglial activation might induce phagocytosis of Aβ, stalling formation of oligomers, and restricting neurotoxicity from deposited Aβ in plaques, while further inflammatory activation might accelerate neurodegeneration. If supported, this distinction could aid patient stratification and guide intervention trials that include immune modification components.

Glial activation occurs as part of altered immune cytokine activities, which also change towards increased inflammatory activity during AD progression. However, micro- and astroglial activation are interlinked, and genetic evidence suggests that innate immunity could be a prime mover in the AD cascade [9]. Based on the described findings of early microglial activation, our starting point was to investigate these events in CSF samples via soluble TREM2 (sTREM2) as a microglial activation marker, and clusterin and chitinase-3-like protein 1 (YKL-40) which both are suggestive of astroglial activation, a marker for neuron-microglia communication (chemokine ligand 1; CX3CL1; fractalkine) and a well-established marker for microglial mobilization and inflammatory reaction (monocyte chemoattractant protein 1, MCP-1).

Soluble TREM2 is released upon microglial activation, leading to increased levels of CSF sTREM2 in AD [29, 30]. This receptor might subserve Aβ uptake by peptides being bound to its ligands APOE and clusterin [31–34]. Clusterin is abundantly expressed by astrocytes and select neuronal populations, e.g., within the hippocampus, and may modulate Aβ metabolism as a chaperone protein [35]. In binding Aβ, clusterin may increase clearance and inhibit plaque formation in processes that are coupled to immune responses [35–37]. YKL-40 is produced mainly by astrocytes, but also microglia, often surrounding amyloid plaques. While early expression levels vary, increased expression has been reported at the MCI stage associated with neuroinflammation [38, 39]. Experimental data suggest a role for YKL-40 in microglia-astroglia crosstalk [38, 40]. Fractalkine is a CXC chemokine (CX3CL1) that is highly expressed by neurons in the hippocampus and cortex, while its receptors (CX3CR1) are found on microglia [41]. Fractalkine neuron-to-microglia communication strengthens the neuroprotective role of microglia, by inhibiting TNFα secretion [42], reducing neurotoxicity, and reducing microglial activation [43, 44]. The expression level of fractalkine has been reported to reflect progression of AD [45]. MCP-1 is a CC chemokine produced by micro- and astroglia and endothelial cells with receptors (CCR2) largely restricted to immune cells but also found on neurons. In the brain, MCP-1 attracts microglial and peripheral immune cells to sites of inflammation. It may stimulate microglia to change from resting to activated morphology, and the level of CSF MCP-1 increases with advancing pathology in AD [46].

These individual markers have been studied in predementia and in AD dementia stages with variable reported findings (see Additional file 1: Table S1). To our knowledge, none of the included CSF immune markers (sTREM2, MCP-1, YKL-40, fractalkine, and clusterin) have been studied in a defined SCD group; however, both CSF sTREM2 and YKL-40 have been studied in preclinical AD (SCD cases and asymptomatic subjects) with pathological (low) CSF Aβ. Neither sTREM2 nor YKL-40 was reportedly increased in this mixed group [29, 30, 38, 40]. Clusterin and fractalkine have been little studied in MCI [47, 48], but sTREM2, MCP-1, and YKL-40 have all shown contradictory results, either unchanged [49–52] or increased [29, 30, 38, 40, 53, 54] compared to controls. Except for YKL-40 [38, 51] and fractalkine [47] which respectively have been found unchanged or reduced compared to controls, all the other immune markers have shown contradictory results in AD dementia compared to controls, either unchanged [29, 49, 51, 55–59], reduced [60, 61], or increased CSF values [38, 50, 53, 54, 62–65].

Intrathecal levels of glial- and inflammation markers may reflect both CNS AD pathogenic processes and responsivity, as well as inflammatory reactivity upon stimulation such as therapeutic interventions and infectious agents. To our knowledge, CSF sTREM2, MCP-1, YKL-40, clusterin, and fractalkine have never been analyzed in the same cohorts across predementia AD stages. Thus, we currently lack information on putative disparate or concerted micro- and astroglial patterns of activation and inflammation related to clinical and neuropathological changes in predementia AD. As activation may be bi- or multiphasic along the AD continuum, highly standardized protocols and measurements on standardized platforms, tightly controlled clinical staging, and biomarker-based stratification may be necessary to detect relevant differences.

Microglial activation per se does not need to be inflammatory, but may be a compensatory response at the synapse. Following Fan et al. [26], we hypothesize that the earliest stage of demonstrable microglial activation occurs at the pre-clinical stage, only coincident with other inflammatory and astroglial activation markers at later stages. We also explore relations between CSF biomarker-derived A/T/N stages and glial activation markers.

## Methods

### Subjects

For the purposes of the present study, we selected 121 participants from two Norwegian cohorts. Healthy controls with normal CSF (*n* = 36), participants with SCD (*n* = 18), and MCI (*n* = 20) patients, both with CSF Aβ42 confirmed amyloid pathology, were selected from the Norwegian multicenter study, “Dementia Disease Initiation” (DDI) [7]. A patient group meeting the National Institute on Aging–Alzheimer’s Association (NIA-AA) criteria for dementia due to AD [6] (*n* = 27) and an additional 19 MCI patients with CSF Aβ42 confirmed amyloid pathology were included from the Norwegian part of the Gothenburg-Oslo MCI (MCI-GO) cohort [66]. Classification of A/T/N groups [1] was done using CSF Aβ42 (A), phosphorylated tau (P-tau) (T), and total-Tau (T-tau) (N). All subjects were assigned binary scores for each category, rated positive when the CSF biomarker value was defined as pathological. The cut-off for CSF was Aβ42 < 708 pg/ml for amyloid plaque pathology, subsequently denoted Aβ-positive (Aβ+) and A+ cases. This Aβ optimal cut-off was determined by a PET [^18^F]-Flutemetamol uptake study [67]. Cases with Aβ42 values close to cutoff (± 30 ng/ml) were excluded from this study material. The abnormality cut-off values for CSF T-tau and P-tau were set in accordance with reference values from Sjögren et al. [68]. For P-tau, the cut-off value was ≥ 80 pg/ml, and values above this threshold were classified as a T+ score. For T-tau, cut-off values were > 300 pg/ml for age < 50 years, > 450 pg/ml for age 50–69 years, and > 500 pg/ml for age ≥ 70 years. Subjects were denoted N+ cases when their T-tau value exceeded the respective thresholds.

Further criteria for inclusion were age between 40 and 80 years and a native language of Norwegian, Swedish, or Danish. Exclusion criteria were brain trauma or disorders, including clinical stroke, dementia, severe psychiatric disorder, and severe somatic disease that might influence the cognitive functions, intellectual disability, or other developmental disorders.

Both DDI and MCI-GO employ a standardized protocol for participant selection, assessment, and disease stage classification according to published criteria [3, 4, 6]. All patients were interviewed and examined by a physician trained in diagnosing cognitive disorders. They all underwent cognitive testing, either cerebral MRI or CT, blood screening, and standard lumbar puncture as part of the clinical assessment.

### Classification of SCD and MCI

Participants were classified as SCD according to the SCD-I framework, which requires normal objective cognitive performance on neuropsychological tests while experiencing a subjective decline in any cognitive domain [3]. MCI was classified according to the NIA-AA criteria which require the presence of subjective cognitive impairment or decline in combination with lower performance than expected in one or more cognitive domains, yet preserved independence in functional ability and not fulfilling the criteria of dementia [4, 6]. Performance was classified as normal or abnormal according to published norms (adjusted for age, sex, and educational effects) for the different tests [69–71]. Due to mutually exclusive criteria, the cut-off values for SCD vs. MCI (defined as normal or abnormal cognition) were ≤ 1.5 standard deviation below normative mean on either Consortium to Establish a Registry for AD (CERAD) word list (delayed recall), Visual Object and Space Perception (VOSP) silhouettes, Trail Making Test part B (TMT-B), or Controlled Oral Word Association Test (COWAT). For the DDI cohort global cognitive status was also assessed by the Clinical Dementia Rating Scale (CDR), whereas the Global Deterioration Scale was used for MCI-GO [72, 73].

### CSF collection and handling

Lumbar punctures were performed similarly on four sites all following a detailed BIOMARKAPD SOPs as described previously [8]. Briefly described, sampling was done before noon and CSF was collected in polypropylene tubes (Thermo Fisher Scientific, MA, USA) which were centrifuged within 4 h at 2000*g* for 10 min at room temperature. The supernatant was subsequently transferred to new defined tubes, directly frozen at − 80 °C on site and kept at − 80 °C until thawed for analysis. All CSF samples were analyzed either at the Department of Interdisciplinary Laboratory Medicine or Section of Clinical Molecular Biology (EpiGen) at Akershus University Hospital. The exception was the sTREM2 analysis, which was assayed at the Department of Pharmacology at the University of Oslo.

### Protein biomarker measurements

Commercial enzyme-linked immunosorbent assays (ELISAs) based on monoclonal antibodies were used to measure CSF levels of the following protein biomarkers: Aβ42, T-tau, and P-tau. They were determined using Innotest β-Amyloid (1-42), Innotest T-tau Ag, and Innotest P-tau (181P)(Fujirebio, Ghent, Belgium), respectively.

CSF sTREM2 was also analyzed using a sandwich ELISA as described earlier [49] with some modifications; the plates were coated over night with the capture antibody (0.25 μg/ml; AF1828, R&D Systems, MN, USA) and samples incubated for 2 h prior to TREM2 detection with a rabbit-monoclonal anti-human TREM2 antibody (0.5 μg/ml; SEK11084, Sino Biologics, Beijing, China).

The QuickPlex SQ 120 system from Meso Scale Discovery (MSD, MD, USA) was used to measure YKL-40, MCP-1, and fractalkine in a U-plex format and clusterin in an R-plex format, where YKL-40 and clusterin were in a singleplex setup and MCP-1 and fractalkine were in the same multiplex setup. The MSD analyses were carried out according to the manufacturers’ procedures, with the adjustments that CSF samples were diluted 200 times prior to YKL-40 and clusterin analyses, and the multiplex setup was used with 100 μl neat CSF and 25 μl buffer.

All the lower limits of quantifications (LLOQs) were defined as the lowest concentration at which the coefficient of variation (CV) of the calculated concentration was < 20% in > 75% of the analyses or the mean CV was < 20% in our test set. All biomarker values in all samples were well above LLOQ. All samples were analyzed in duplicates and reanalyzed if relative deviations (RDs) exceeded 20%. In addition, quality control samples with RD threshold of 15% assured inter-plate and inter-day variation.

### Statistical analysis

Normality was assessed through the inspection of Q-Q plots, histograms, and the Shapiro-Wilks test of normality.

In order to explore and adjust for age and sex, and APOE-ɛ4 allelic effects on CSF inflammatory markers in healthy aging, simple and multiple regression analyses (controlling for several covariates) were performed between these variables and CSF immune markers within the healthy control group. If a significant relationship was observed between these covariates and an inflammatory marker in the healthy control group, the standardized residuals from the pertinent regression model was obtained for the entire sample and used in further analysis in order to adjust for these covariates in between-group comparisons. To assess differences in biomarker levels between groups, we performed one-way ANOVAs with planned comparisons for variables with normal distributions. We performed Kruskal-Wallis test with Dunn’s non-parametric pairwise post hoc test with Bonferroni corrections to assess group differences in variables with non-normal distributions (CSF Aβ42, CSF T-tau, CSF P-tau, MMSE, and A/T/N groups). Non-parametric pairwise comparisons and ANOVA contrasts were performed in a hierarchical manner. We compared Aβ + SCD, MCI, and AD dementia groups to healthy controls, and finally we compared the SCD with the MCI group and both SCD and MCI to the AD dementia group. The dichotomous variable “sex” was assessed using a chi-square test. For the A/T/N groups, A−T−N− and A+T−N− were compared to all other groups. Only one patient had A+T+N− classification, and this patient was excluded from both statistical analysis and figure.

To assess clinical stage dependent relationships between the innate immune response to AD pathology, correlational analyses between the inflammatory markers (sTREM2, YKL40, MCP-1, fractalkine, and clusterin) and CSF AD biomarkers (Aβ42, T-Tau, and P-tau) were performed using Pearson’s *r* within the pertinent symptomatic groups (SCD, MCI, and AD dementia).

All analyses were performed in the Statistical Package for Social Sciences (SPSS) version 25, and the significance level was defined as *p* < .05.

### Ethics

The regional medical research ethics committee approved this study. Participants gave their written informed consent before taking part in the study. All further study conduct was in line with the guidelines provided by the Declaration of Helsinki (1964; revised 2013) and the Norwegian Health and Research Act (2009).

## Results

See Table 1 for further characterization of the study cohort.The mean concentration and standard deviations for sTREM2, MCP-1, YKL-40, fractalkine, and clusterin at the different clinical stages are shown in Table 2.

**Table 1:** Demographic characteristics, AD biomarkers, and ATN groups of the participants by diagnostic category

Variable	Groups				*X*^*2*^(*p*)	Dunn’s pair-wise comparisons					
	1. Healthy controls (*n* = 36)	2. Aβ + SCD (*n* = 18)	3. Aβ + MCI (*n* = 40)	4. AD dementia (*n* = 27)		1 vs 2	1 vs 3	1 vs 4	2 vs 3	2 vs 4	3 vs 4
Age	61.1 (9.2)	67.2 (6.6)	66.6 (7.4)	67.6 (5.2)	*X*^*2*^ = 14.4 (< .01)	n.s.	< .05	< .05	n.s.	n.s.	n.s.
Female %	19 (53%)	8 (44%)	23 (57%)	13 (48%)	*X*^*2*^ = 1.6 (n.s.)	^a^	^a^	^a^	^a^	^a^	^a^
MMSE	29.4 (0.7)	29.2 (0.8)	27.9 (2.0)	19.0 (5.8)	*X*^*2*^ = 83.7 (< .001)	n.s.	< .001	< .001	< .01	< .001	< .001
*APOE*-ɛ4 positivity (%)	16 (44%)	13 (72%)	23 (57%)	9 (33%)	*X*^*2*^ = 6.8 (n.s.)	^a^	^a^	^a^	^a^	^a^	^a^
CSF Aβ42	1044 (185)	530 (98)	474 (112)	473 (93)		^b^	^b^	^b^	^b^	^b^	^b^
CSF Total tau	298 (97)	487 (249)	635 (290)	961 (417)	*X*^*2*^ = 58.0 (< .001)	< .05	< .001	< .001	n.s.	< .01	< .05
CSF P-tau	51 (13)	74 (33)	86 (40)	89 (43)	*X*^*2*^ = 37.2 (< .001)	< .05	< .001	< .001	n.s.	n.s.	n.s.
A−T−N−, *n* (%)	36 (100%)	0 (0%)	0 (0%)	0 (0%)		^b^	^b^	^b^	^b^	^b^	^b^
A+T−N−, *n* (%)	0 (0%)	10 (55%)	13 (33%)	0 (0%)		^b^	^b^	^b^	^b^	^b^	^b^
A+T−N+, *n* (%)	0 (0%)	1 (5%)	10 (25%)	19 (70%)		^b^	^b^	^b^	^b^	^b^	^b^
A+T+N+, *n* (%)	0 (0%)	7 (40%)	16 (40%)	8 (30%)		^b^	^b^	^b^	^b^	^b^	^b^

Abbreviations: *n.s*. non-significant, Aβ*+* indicates CSF Aβ42 below the normal range

^a^Non-parametric post hoc analysis not performed due to non-significant Kruskal-Wallis test. All *p* values are Bonferroni corrected

^b^ No statistical tests applied

**Table 2:** Between-group CSF inflammation marker comparisons

Variable	Groups				*F*(*p*)	ANOVA contrasts (*p*)					
	1. Healthy controls (*n* = 36)	2. Aβ + SCD (*n* = 18)	3. Aβ + MCI (*n* = 40)	4. AD dementia (*n* = 27)		1 vs 2	1 vs 3	1 vs 4	2 vs 3	2 vs 4	3 vs 4
CSF sTREM2	3.1 (0.9)	4.6 (1.9)	4.0 (1.8)	4.8 (1.7)	*F* = 7.0 (*p* < .001)	< .01^b^	< .05^b^	< .001^b^	n.s. ^b^	n.s. ^b^	< .05^b^
CSF MCP-1	483 (124)	510 (133)	581 (160)	645 (249)	*F* = 5.3 (*p* < .01)	n.s.^b^	< .05^b^	< .001^b^	n.s.^b^	< .05^b^	n.s.^b^
CSF YKL-40	145 (46)	188 (80)	182 (69)	221 (70)	*F* = 3.4 (*p* < .05)	n.s. ^b^	n.s. ^b^	< .01^b^	n.s. ^b^	n.s. ^b^	< .05^b^
CSF fractalkine	1823 (446)	1924 (461)	1790 (469)	1983 (569)	*F* = 1.0 (*p* = n.s.)	^a^	^a^	^a^	^a^	^a^	^a^
CSF clusterin	2286 (828)	2305 (719)	2611 (817)	2777 (769)	*F* = 2.6 (*p* = n.s.)	^a^	^a^	^a^	^a^	^a^	^a^

Abbreviations: *n.s.* non-significant (*p* > .05), Aβ+ indicates CSF Aβ42 below the normal range

^a^Contrasts not performed due to non-significant ANOVA

^b^Equal distribution assumed

### Age, sex, and APOE-ɛ4 allelic relationship with CSF immune markers in healthy controls

Neither APOE-ɛ4 status nor sex was associated with any of the CSF inflammatory markers in healthy controls when using simple regression analysis. When age and CSF T-tau were included in multiple regression models, both age (*β* = .477, *p* < .001) and T-tau (*β* = .384, *p* < .01) were shown to predict CSF YKL-40 (adjusted *R*^2^ = .497, *F*[2, 33] = 18.280, *p* < .001) (Additional file 2). The same was not true for CSF sTREM2, where the relationship between age and sTREM2 was not found in healthy controls (*β* = .248, *p* = n.s.), when controlling for the effects of T-tau (*β* = .478, *p* < .01) in a multiple regression analysis (adjusted *R*^*2*^ = .342, *F*[2, 32] = 9819, *p* < .001). No relationships were found between age and CSF MCP-1, clusterin, or fractalkine within the healthy control group in any regression models. Thus, only YKL-40 was adjusted for age-effect prior to analysis of between-group differences in clinical staging.

### CSF activation and inflammation marker comparisons based on clinical staging

**Fig. 1:**
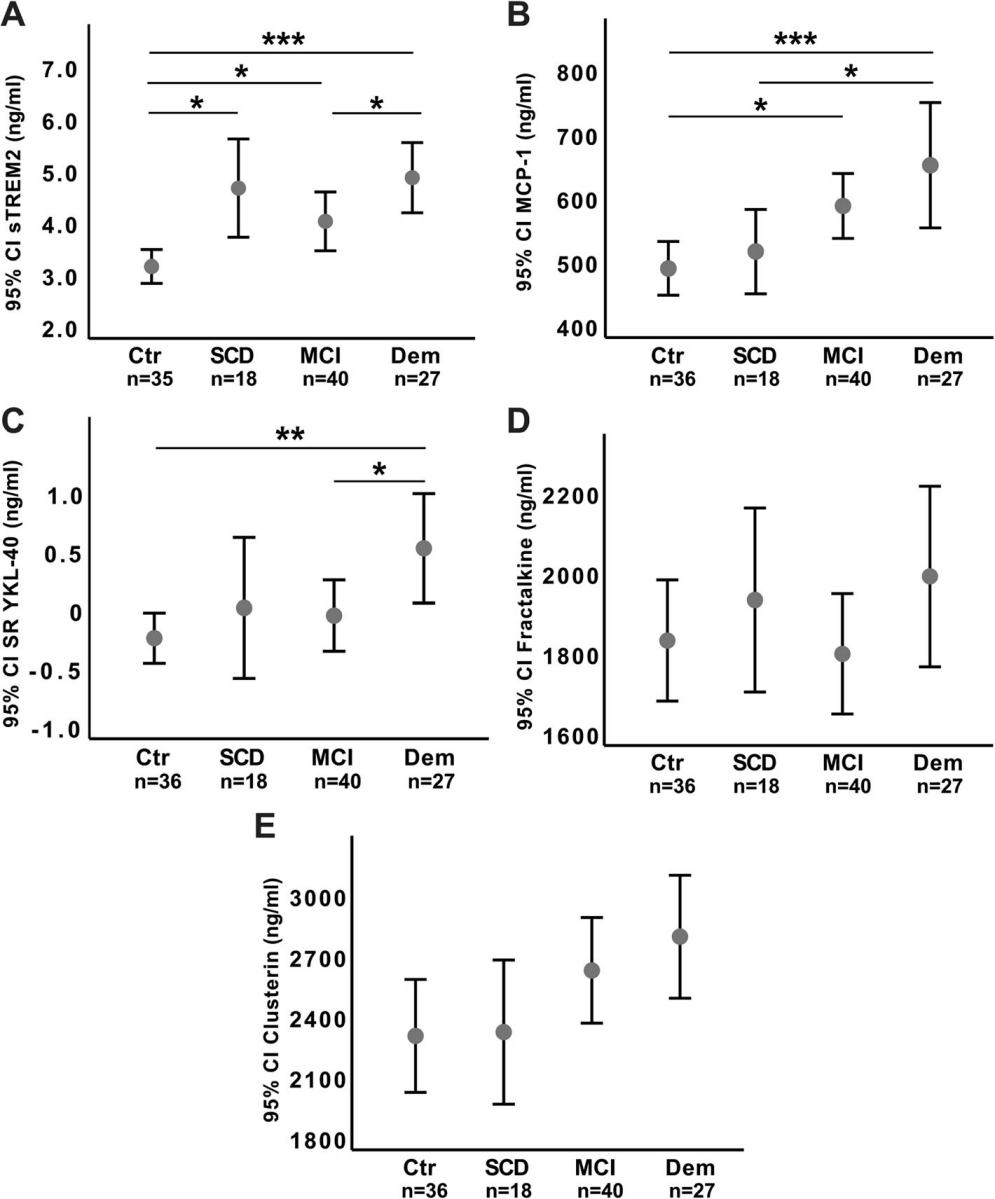
Between-group CSF immune marker comparisons based on clinical staging. Fig. text: The *Y*-axis with sTREM2 (**a**), MCP-1 (**b**), fractalkine (**d**), and clusterin (**e**) reported as CSF concentration in nanograms per milliliter, while the *Y*-axis for YKL-40 (**c**) are residuals standardized for age. Error bars are shown as mean and 95% confidence interval (CI). Abbreviation: Ctr: healthy controls (*n* = 36), SCD: CSF Aβ42+ subjects with subjective cognitive decline (*n* = 19), MCI: CSF Aβ42+ subjects with mild cognitive impairment (*n* = 39), Dem: Aβ42+ subjects with Alzheimer’s disease dementia (*n* = 27). Statistically significant differences are marked with asterisks, where * indicates *p* < .05, ** indicates *p* < .01, and *** indicates *p* < .001

CSF level comparisons between clinical groups are summarized in Table 2, illustrated in Fig. 1, and further compared in Additional file 3: Figure S2. CSF sTREM2, YKL-40, and MCP-1 were increased at more advanced clinical stages, but differed according to which cognitive stage they showed abnormal levels.

CSF sTREM2 values were higher in Aβ + SCD subjects (*t*(116) = 3.282, *p* < .01), Aβ + MCI subjects (*t*(116) = 2.364, *p* < .05), and subjects with AD dementia (*t*(116) = 4.213, *p* < .001), compared to healthy controls. A higher CSF sTREM2 level was also found in subjects with AD dementia compared to Aβ + MCI subjects (*t*(116) = 2.135, *p* < .05).

No difference in the CSF YKL-40 level was found between healthy controls, and Aβ + SCD or Aβ + MCI subjects. However, an increased level of CSF YKL-40 was found in subjects with AD dementia compared to both Aβ + MCI subjects (*t*(117) = 2.370, *p* < .05) and healthy controls (*t*(117) = 3.096, *p* < .01).

No differences in CSF MCP-1 levels were demonstrated between Aβ + SCD and healthy controls. However, CSF MCP-1 levels were equally increased in Aβ + MCI subjects and subjects with AD dementia, and levels where higher in these groups compared to healthy controls ((*t*(117) = 2.480, *p* < .05) and (*t*(117) = 3.704, *p* < .001) respectively).

Finally, no significant differences between clinical groups were found for either CSF fractalkine or CSF clusterin. The between-group ANOVA analysis for clusterin was, however, borderline significant (*F*(3, 117) = 2.574, *p* = .057), with markedly higher CSF clusterin levels in the dementia group compared to the control group (Fig. 1).

### CSF activation and inflammation marker comparisons based on A/T/N biomarker classification

**Fig. 2:**
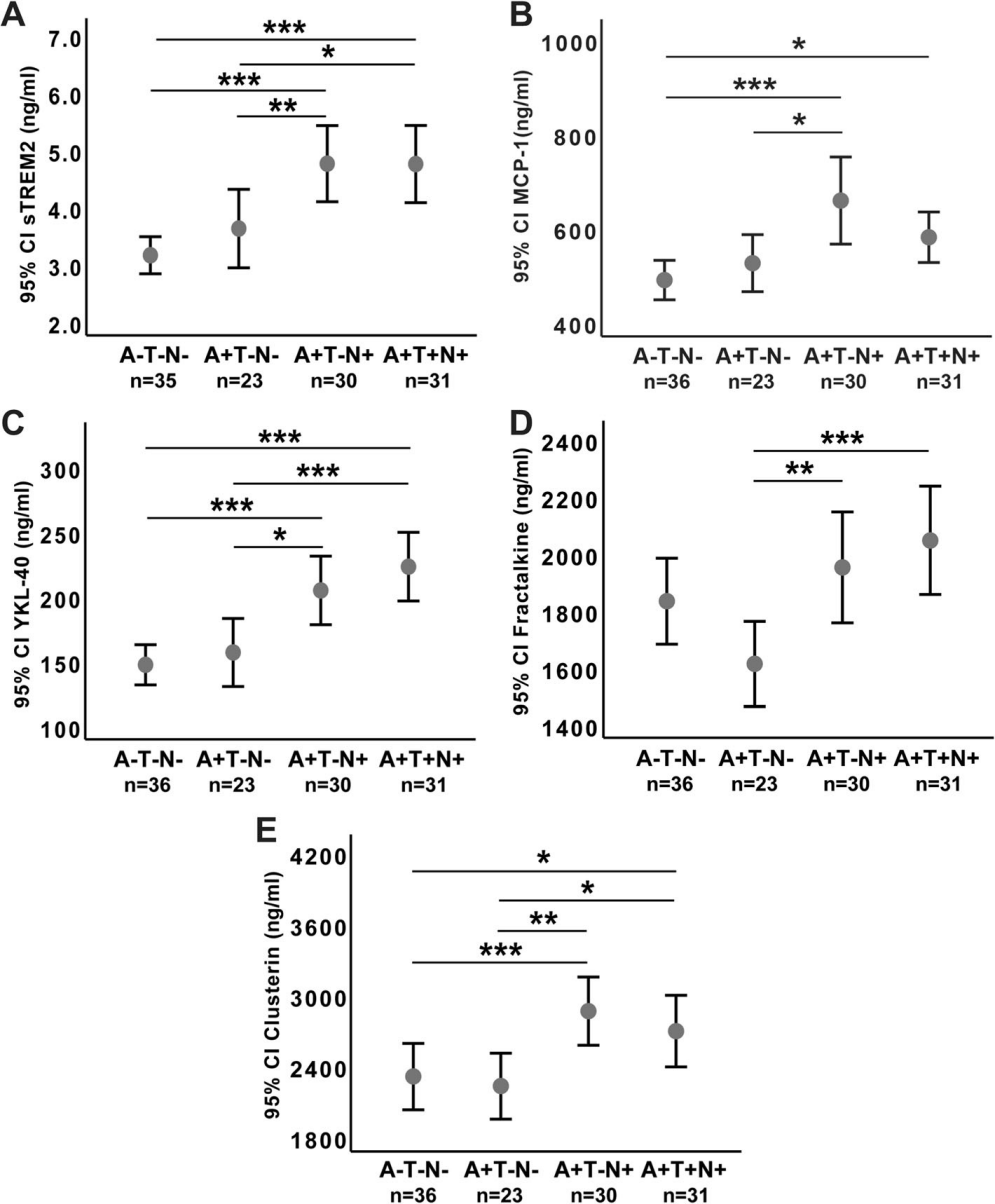
Between-group CSF immune marker comparisons based on ATN staging. Fig. text: *The association with ATN groups. Y*-axis shows concentration of inflammatory markers in CSF in nanograms per milliliter. A+ indicating CSF Aβ42 below the reference range, T+ indicating CSF p-tau above the reference range and N+ here indicating T-tau above the reference range for age. Minus (−) indicating normal values within the reference range. Error bars are shown as mean and 95% confidence interval (CI). Statistically significant differences are marked with asterisks, where * indicates *p* < .05, ** indicates *p* < .01, and *** indicates *p* < .001

CSF level comparisons between A/T/N groups are illustrated in Fig. 2.

No differences in CSF sTREM2, YKL-40, MCP-1, or CSF clusterin levels were found between healthy controls with normal CSF (A−T−N− *n* = 36) compared to participants with cognitive symptoms with A+T−N− (*n* = 23). However, the levels of these markers were all equally increased in A+T−N+ (*n* = 30) and A+T+N+ (*n* = 31), *p* < .01–*p* < .001 and *p* < .05–*p* < 01 respectively, please see Table 1 and Fig. 2 for details. Within the SCD group, there were no significant differences between those with pathological levels of T-tau and/or P-tau (*n* = 9) and those with normal CSF levels, for either CSF sTREM2, YKL-40, MCP-1, clusterin, or fractalkine (independent samples *T*-test), nor no significant correlations between CSF MCP-1 or clusterin and T-tau or P-tau. However, there were significant positive correlations between CSF sTREM2, YKL-40, and fractalkine with both T-tau and P-tau within the SCD group (Table 3).

**Table 3:** Correlation analyses between inflammatory and AD CSF biomarkers by diagnostic category

All symptomatic subjects (SCD, MCI, AD dementia) (*n* = 85)					
**Variable**	**CSF sTREM2**	**CSF YKL-40**	**CSF MCP-1**	**CSF Fractalkine**	**CSF Clusterin**
CSF Aβ42	n.s.	n.s.	n.s.	n.s.	n.s.
CSF T-tau	.318, *p* < .01	.589, *p* < .001	.276, *p* < .05	.507, *p* < .001	.400, *p* < .001
CSF P-tau	.289, *p* < .01	.444, *p* < .001	n.s.	.368, *p* < .001	.290, *p* < .01
SCD (*n* = 18)					
**Variable**	**CSF sTREM2**	**CSF YKL-40**	**CSF MCP-1**	**CSF Fractalkine**	**CSF Clusterin**
CSF Aβ42	n.s.	n.s.	n.s.	n.s.	.568, *p* < .05
CSF T-tau	.629, *p* < .01	.730, *p* < .001	n.s.	.616, *p* < .01	n.s.
CSF P-tau	.655, *p* < .01	.679, *p* < .01	n.s.	.536, *p* < .05	n.s.
MCI (*n* = 40)					
**Variable**	**CSF sTREM2**	**CSF YKL-40**	**CSF MCP-1**	**CSF Fractalkine**	**CSF Clusterin**
CSF Aβ42	n.s.	n.s.	n.s.	n.s.	n.s.
CSF T-tau	n.s.	.409, *p* < .01	n.s.	.479, *p* < .01	.330, *p* < .05
CSF P-tau	.335, *p* < .05	.347, *p* < .05	n.s.	.375, *p* < .05	.364, *p* < .05
AD dementia (*n* = 29)					
**Variable**	**CSF sTREM2**	**CSF YKL-40**	**CSF MCP-1**	**CSF Fractalkine**	**CSF Clusterin**
CSF Aβ42	n.s.	n.s.	n.s.	n.s.	n.s.
CSF T-tau	n.s.	.660, *p* < .001	n.s.	.550, *p* < .01	.415, *p* < .05
CSF P-tau	n.s.	.439, *p* < .05	n.s.	n.s.	n.s.

Reported values are Pearson’s *r* and associated *p* value

*n.s*. non-significant (*p* > .05)

CSF fractalkine was increased in A+T−N+ (*n* = 30) and A+T+N+ (*n* = 31) compared to patients with pathological CSF Aβ without tau pathology or neurodegeneration markers A+N−T− (*n* = 23) (*p* < .001 and *p* < .01 respectively), in accordance with the association between fractalkine and T-tau (Table 3). CSF fractalkine was lower (1603 ng/ml, SD 345) in A+T−N− than in A−T−N− healthy controls (1823 ng/ml, SD 446), but this difference did not reach statistical significance (*p* = .057). CSF clusterin levels were equally increased in both A+T−N+ (*n* = 30) and A+T+N+ (*n* = 31) compared to healthy controls with normal CSF (A−T−N−, *n* = 36) (*p* < .01 and *p* < .05 respectively) and also in accordance with the association with T-tau (Table 3).

There were no significant correlations between sTREM2, YKL-40, MCP-1, or fractalkine and CSF Aβ42 in our cohort, neither in subgroups controlled separately nor when all symptomatic subjects were combined. There was, however, a correlation between CSF clusterin and CSF Aβ42 (*β* = 0.568, *p* < 0.05) in the SCD group. CSF T-tau correlated strongest with all of the before mentioned activation markers when all symptomatic subjects were combined, in addition to several of the subgroups. For details, please see Table 3.

## Discussion

In this study, we have demonstrated microglial activation in Aβ + SCD cases, as shown by increased sTREM2. Since there were no differences in objective cognitive performance between the healthy controls and the SCD group (see Table 1), increased CSF sTREM2 in the Aβ + SCD group suggests microglial activation even before objective cognitive decline. Furthermore, we did not find evidence for neither astroglial activation at this stage (no significant increase of YKL-40 or clusterin) nor for a microglial inflammatory response (no significant increase in MCP-1 levels). CSF MCP-1 levels were increased in the Aβ + MCI group compared to healthy controls, indicative of an inflammatory response at the MCI stage. This interpretation is corroborated by the increase in astroglial markers at the dementia stage, indicative of involvement of astrocytes in the inflammatory process as loss of cognitive abilities progresses even further. These differences may represent functionally important stages of innate immune activation and neuroinflammation along the AD continuum and are summarized in Additional file 3: Figure S2.

### Increasing inflammation with increasing neurodegeneration, but not Aβ pathology alone

All of the inflammatory markers were increased in CSF in subjects with pathological T-tau (A+T−N+ and A+T+N+), indicating neurodegeneration, compared to those subjects without neurodegeneration (A−T−N− and A+T−N−). Interestingly, neither glial activation markers nor inflammatory markers were significantly increased in cases with only Aβ+ pathology (A+T−N−) compared to healthy controls (A−T−N−), though CSF fractalkine showed a non-significant reduction. For both CSF fractalkine and CSF clusterin, the increase with increasing neurodegeneration may be masked when comparing the clinical groups, as these contain subjects with and without neurodegeneration, but it became evident when we employed the A/T/N classification. For CSF fractalkine, we found no between group differences between the control group/A−T−N− compared to the other clinical groups. However, when comparing A/T/N groups as a measure of AD biomarker severity, the Aβ42-positive group without neurodegeneration (A+T−N−, 13 subjects with MCI and 9 with SCD) showed significantly lower CSF fractalkine levels compared to the Aβ42-positive groups with neurodegeneration (A+T−N+, 1 SCD subject, 10 MCI subjects, and 19 AD dementia, and A+T+N+, 7 SCD subjects, 16 MCI subjects, and 8 AD dementia). CSF fractalkine showed a clear association to T-tau in all clinical subgroups, see Table 3. Moreover, CSF clusterin was also associated with T-tau in both the MCI and dementia subgroups, but not the SCD group. While the between-group ANOVA analysis for clusterin did not reach the threshold for statistical significance, the dementia group did show markedly higher levels compared to the control group. This negative result may have been due to the inclusion of a heterogeneous sample with relatively small subgroups. When our cohort was categorized according to the A/T/N biomarker classification scheme, however, the difference between the groups without (A−T−N− and A+T−N−) and those with neurodegeneration (A+T−N+ and A+T+N+) was clearly evident (Fig. 2). Thus, findings relative to clinical and A/T/N stages are in general accordance, both consistent with a restricted microglial activation accompanying amyloid pathology (A+) without more extensive inflammatory activation, the latter accompanying neurodegeneration, and established cognitive impairment at the MCI and dementia stages.

Our findings in the clinical stages are in accordance with imaging data suggestive of a biphasic microglial response, though the sTREM2 level in our material at the SCD stage was only nominally higher but not significantly different from that at the MCI stage [25, 26]. Aging is associated with subtle microglial priming, facilitating phagocytosis and homeostatic recovery but also further development of potentially detrimental inflammatory properties that may increase Aβ and tau pathologies [74, 75]. A sequence of events is not established, as microglia may be primed by several types of stimuli including Aβ, but our description of the relation between inflammatory and neurodegeneration markers is consistent with the above description.

Our findings may help interpret studies of immunomodulating therapies towards reducing the Aβ deposition by increasing the microglial clearance [76]. A too advanced stage of AD may have contributed to trial failures with several phase III studies being discontinued due to lack of treatment efficacy or side effects. Cerebral Aβ aggregation starts 10 to 15 years before mild cognitive decline [77] and 20 to 30 years before dementia onset [78]. The present findings suggest that the innate immune system and astroglial cells may undergo sequential changes towards an inflammatory activation also during the preclinical part of this period, represented by sTREM2 and YKL-40, respectively. Immunomodulating therapies might have different effects depending on the state of immune activation, i.e., whether monocyte and astroglial inflammatory components are activated or not [26]. Thus, immune activation should be investigated in greater detail by analyzing CSF markers pro- and anti-inflammatory markers throughout the AD continuum, which we plan to explore in a subsequent study.

A limitation of these findings is that these markers are expressed or secreted by multiple cell types and are not completely specific to neuronal, microglial, or astrocyte expression. Fractalkine is mainly expressed on neurons in the CNS [79], but expression is also seen in astrocytes [80], especially in neuroinflammatory models [81–83]. Also, the choroid plexus has been shown to express fractalkine in experimental activation models [84]. MCP-1 is expressed by astrocytes [85, 86], microglia [87] and neurons [88]. YKL-40 is primarily expressed by astrocytes and to a lesser degree by microglia [89]. Although clusterin is mainly expressed by astrocytes [90–93], and especially reactive astrocytes [94], expression has also been shown in neurons [92–95] and degenerating oligodendrocytes [91]. TREM2, on the other hand, seems to be specific to microglia in the brain [96–99]. However, in peripheral tissue, TREM2 has been shown to be expressed by several myeloid cells, including tissue macrophages and dendritic cells [100].

In addition to a relatively small sample size, a further limitation in this work is the cross-sectional design, as longitudinal sampling is necessary for a better interpretation of the sequence of events along the AD continuum. Furthermore, a more extensive mapping of inflammatory cytokine markers will be needed.

## Conclusion

We here demonstrate increased microglial activation at a preclinical AD stage, with increased CSF sTREM2 levels in Aβ + SCD. The MCP-1 level was significantly enhanced at the Aβ + MCI stage, and CSF YKL-40 in AD dementia, suggesting a shift towards a more harmful stage of immune activation as AD progresses. Furthermore, our findings suggest that inflammation is associated with neurodegeneration, but not with amyloid pathology alone.

## Additional files

Additional file 1: **Table S1.** Literature overview CSF immune markers in AD clinical groups. (DOCX 72 kb)

Additional file 2: **Figure S1.** Correlation between CSF YKL-40 and age. (PDF 805 kb)

Additional file 3: **Figure S2.** Between-group comparisons of microglial- and astroglial activation with inflammation based on clinical staging. (PDF 277 kb)

## Abbreviations

AD: Alzheimer’s disease
ANOVA: Analysis of variance
Aβ1-42: Amyloid beta 1-42 peptide
CCR2: CC chemokine receptor type 2, MCP-1 receptor
CDR: Clinical Dementia Rating Scale
CERAD: Consortium to Establish a Registry for Alzheimer’s Disease
COWAT: Controlled Oral Word Association Test
CSF: Cerebrospinal fluid
CT: Computer tomography
CV: Coefficient of variation
CX3CL1: Fractalkine
CX3CR1: Fractalkine receptor
DDI: Dementia Disease Initiation
LLOQ: Lower limit of quantification
MCI: Mild cognitive impairment
MCI-GO: Gothenburg-Oslo MCI
MCP-1: Monocyte chemoattractant protein 1
MMSE: Mini-Mental State Examination
MRI: Magnetic resonance imaging
MSD: Meso Scale Discovery
n.s.: Non-significant
NIA-AA: National Institute on Aging–Alzheimer’s Association
PET: Positron emission tomography
P-tau: Phosphorylated tau
Q-Q plots: Quantile-quantile plots
RD: Relative deviation
SCD: Subjective cognitive decline
SD: Standard deviation
SOP: Standard operating procedure
SPSS: Statistical Package for Social Sciences
sTREM2: Soluble triggering receptor expressed on myeloid cells 2
TMT-B: Trail Making Test part B
T-tau: Total tau
VOSP: Visual Object and Space Perception
YKL-40: Chitinase-3-like protein 1
Aβ+: Pathological amyloid beta 142 peptide in the cerebrospinal fluid
