# Supplementary material for: Glial activation and inflammation along the Alzheimer’s disease continuum
Source: J Neuroinflammation. 2019 Feb 21;16:46. doi: 10.1186/s12974-019-1399-2 (PMC6383268; doi:10.1186/s12974-019-1399-2)
Supplement: Supplementary file 1 — Table S1. Literature overview CSF immune markers in AD clinical groups. (DOCX 72 kb) [file 12974_2019_1399_MOESM1_ESM.docx]

|  | Preclinical | MCI | AD |
| --- | --- | --- | --- |
| sTREM2 | ⇔ Gispert et al., 2016(1)  ⇔ Suarez-Calvet et al., 2016(2)  ⇑ Liu et al., 2018* (3) | ⇔ Henjum et al., 2016 (4)  ⇔ Lie et al., 2018* (3)  ⇑ Suarez-Calvet et al., 2016(2)  ⇑ Gispert et al., 2016(1)  ⇑ Brosseron et al., 2018(5) | ⇔ Henjum et al., 2016 (4)  ⇔ Suarez-Calvet et al., 2016(2)  ⇓ Kleinberger et al., 2014 (6)  ⇑ Gispert et al., 2016 (7)  ⇑ Heslegrave et al., 2016(8)  ⇑ Piccio et al., 2016 (9)  ⇑ Liu et al., 2018* (3)  ⇑ Brosseron et al., 2018(5) |
| MCP-1 | *Not studied* | ⇔ Mattsson et al., 2011 (10)  ⇔ Westin et al., 2012 (11)  ⇑ Galimberti et al., 2006 (12) | ⇔ Mattsson et al., 2011 (10)  ⇔ Blasko et al., 2006 (13)  ⇑ Galimberti et al., 2006 (12) |
| YKL-40 | ⇔ Antonell et al., 2014 (14)  ⇔ Gispert et al 2016(7) | ⇔ Mattsson et al., 2011(10)  ⇑ Antonell et al., 2014 (14)  ⇑ Gispert et al 2016(7) | ⇔ Gispert et al., 2016 (7)  ⇔ Mattsson et al., 2011(10) |
| Fractalkine | *Not studied* | ⇔ Perea et al., 2018(15) | ⇓ Perea et al., 2018(15) |
| Clusterin | *Not studied* | ⇔ Desikan et al., 2014(16) | ⇔ Lindstrom et al., 2001 (17)  ⇔ Vranova et al., 2016 (18)  ⇔ Richens et al., 2014(19)  ⇔ Yang et al., 2018 (20)  ⇑ Deming et al., 2016 (21)  ⇑ Nilselid et al., 2006 (22)  ⇓ Puchades et al., 2003 (23) |
| ⇑ increased, ⇔ unchanged or ⇓ reduced as compared to healthy controls. *metanalysis | | | |

1. Gispert JD, Suarez-Calvet M, Monte GC, Tucholka A, Falcon C, Rojas S, et al. Cerebrospinal fluid sTREM2 levels are associated with gray matter volume increases and reduced diffusivity in early Alzheimer's disease. Alzheimer's & dementia : the journal of the Alzheimer's Association. 2016;12(12):1259-72.

2. Suarez-Calvet M, Kleinberger G, Araque Caballero MA, Brendel M, Rominger A, Alcolea D, et al. sTREM2 cerebrospinal fluid levels are a potential biomarker for microglia activity in early-stage Alzheimer's disease and associate with neuronal injury markers. EMBO Mol Med. 2016;8(5):466-76.

3. Liu D, Cao B, Zhao Y, Huang H, McIntyre RS, Rosenblat JD, et al. Soluble TREM2 changes during the clinical course of Alzheimer's disease: A meta-analysis. Neurosci Lett. 2018;686:10-6.

4. Henjum K, Almdahl IS, Arskog V, Minthon L, Hansson O, Fladby T, et al. Cerebrospinal fluid soluble TREM2 in aging and Alzheimer's disease. Alzheimers Res Ther. 2016;8(1):17.

5. Brosseron F, Traschutz A, Widmann CN, Kummer MP, Tacik P, Santarelli F, et al. Characterization and clinical use of inflammatory cerebrospinal fluid protein markers in Alzheimer's disease. Alzheimers Res Ther. 2018;10(1):25.

6. Kleinberger G, Yamanishi Y, Suarez-Calvet M, Czirr E, Lohmann E, Cuyvers E, et al. TREM2 mutations implicated in neurodegeneration impair cell surface transport and phagocytosis. Sci Transl Med. 2014;6(243):243ra86.

7. Gispert JD, Monte GC, Falcon C, Tucholka A, Rojas S, Sanchez-Valle R, et al. CSF YKL-40 and pTau181 are related to different cerebral morphometric patterns in early AD. Neurobiol Aging. 2016;38:47-55.

8. Heslegrave A, Heywood W, Paterson R, Magdalinou N, Svensson J, Johansson P, et al. Increased cerebrospinal fluid soluble TREM2 concentration in Alzheimer's disease. Mol Neurodegener. 2016;11:3.

9. Piccio L, Deming Y, Del-Aguila JL, Ghezzi L, Holtzman DM, Fagan AM, et al. Cerebrospinal fluid soluble TREM2 is higher in Alzheimer disease and associated with mutation status. Acta Neuropathol. 2016;131(6):925-33.

10. Mattsson N, Tabatabaei S, Johansson P, Hansson O, Andreasson U, Mansson JE, et al. Cerebrospinal fluid microglial markers in Alzheimer's disease: elevated chitotriosidase activity but lack of diagnostic utility. Neuromolecular Med. 2011;13(2):151-9.

11. Westin K, Buchhave P, Nielsen H, Minthon L, Janciauskiene S, Hansson O. CCL2 is associated with a faster rate of cognitive decline during early stages of Alzheimer's disease. PLoS One. 2012;7(1):e30525.

12. Galimberti D, Schoonenboom N, Scheltens P, Fenoglio C, Bouwman F, Venturelli E, et al. Intrathecal chemokine synthesis in mild cognitive impairment and Alzheimer disease. Arch Neurol. 2006;63(4):538-43.

13. Blasko I, Lederer W, Oberbauer H, Walch T, Kemmler G, Hinterhuber H, et al. Measurement of thirteen biological markers in CSF of patients with Alzheimer's disease and other dementias. Dement Geriatr Cogn Disord. 2006;21(1):9-15.

14. Antonell A, Mansilla A, Rami L, Llado A, Iranzo A, Olives J, et al. Cerebrospinal fluid level of YKL-40 protein in preclinical and prodromal Alzheimer's disease. J Alzheimers Dis. 2014;42(3):901-8.

15. Perea JR, Lleo A, Alcolea D, Fortea J, Avila J, Bolos M. Decreased CX3CL1 Levels in the Cerebrospinal Fluid of Patients With Alzheimer's Disease. Front Neurosci. 2018;12:609.

16. Desikan RS, Thompson WK, Holland D, Hess CP, Brewer JB, Zetterberg H, et al. The role of clusterin in amyloid-beta-associated neurodegeneration. JAMA neurology. 2014;71(2):180-7.

17. Lidstrom AM, Hesse C, Rosengren L, Fredman P, Davidsson P, Blennow K. Normal levels of clusterin in cerebrospinal fluid in Alzheimer's disease, and no change after acute ischemic stroke. J Alzheimers Dis. 2001;3(5):435-42.

18. Prikrylova Vranova H, Henykova E, Mares J, Kaiserova M, Mensikova K, Vastik M, et al. Clusterin CSF levels in differential diagnosis of neurodegenerative disorders. J Neurol Sci. 2016;361:117-21.

19. Richens JL, Vere KA, Light RA, Soria D, Garibaldi J, Smith AD, et al. Practical detection of a definitive biomarker panel for Alzheimer's disease; comparisons between matched plasma and cerebrospinal fluid. Int J Mol Epidemiol Genet. 2014;5(2):53-70.

20. Yang C, Wang H, Li C, Niu H, Luo S, Guo X. Association between clusterin concentration and dementia: a systematic review and meta-analysis. Metab Brain Dis. 2018.

21. Deming Y, Xia J, Cai Y, Lord J, Holmans P, Bertelsen S, et al. A potential endophenotype for Alzheimer's disease: cerebrospinal fluid clusterin. Neurobiol Aging. 2016;37:208.e1-.e9.

22. Nilselid AM, Davidsson P, Nagga K, Andreasen N, Fredman P, Blennow K. Clusterin in cerebrospinal fluid: analysis of carbohydrates and quantification of native and glycosylated forms. Neurochem Int. 2006;48(8):718-28.

23. Puchades M, Hansson SF, Nilsson CL, Andreasen N, Blennow K, Davidsson P. Proteomic studies of potential cerebrospinal fluid protein markers for Alzheimer's disease. Brain Res Mol Brain Res. 2003;118(1-2):140-6.
