# Supplementary material for: Glial activation and inflammation along the Alzheimer’s disease continuum
Source: J Neuroinflammation. 2019 Feb 21;16:46. doi: 10.1186/s12974-019-1399-2 (PMC6383268; doi:10.1186/s12974-019-1399-2)
Supplement: Supplementary file 2 — Figure S1. Correlation between CSF YKL-40 and age. (PDF 805 kb) [file 12974_2019_1399_MOESM2_ESM.pdf]

Supplementary figure 1: Correlation between CSF YKL-40 and age.

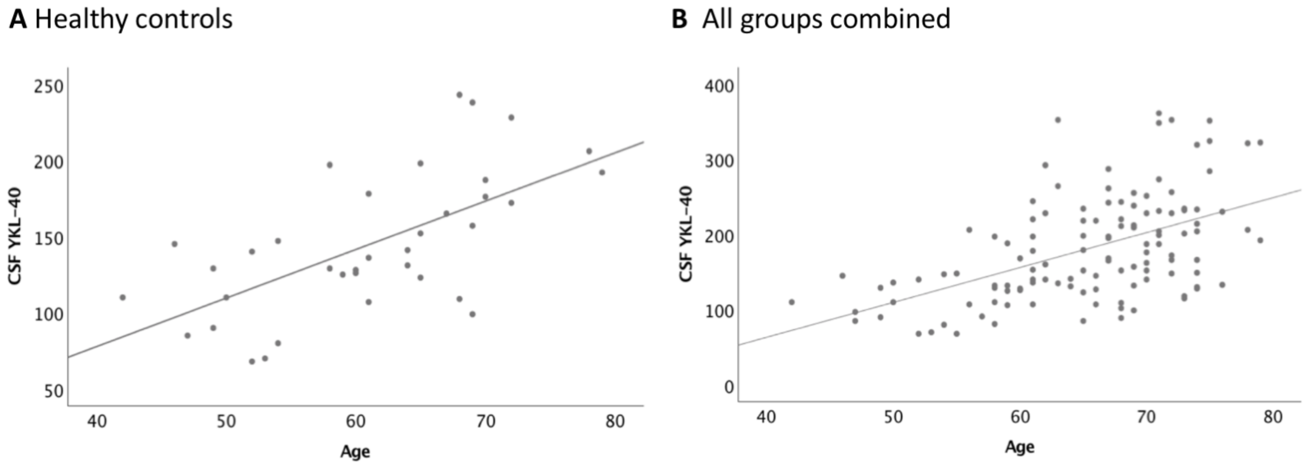

CSF YKL-40 (ng/ml) is correlated to age in healthy controls (A) and all groups combined (B). In simple linear regression CSF YKL-40 show a strong correlation with age among healthy controls (R2 = .403, F(1,34)=22.955, *p*<.001) and the combined cohort consisting of healthy controls, SCD, MCI and AD dementia (R2 = .272, F(1,119)=44.527, *p*<.001), visualized here as a scatter plot.
