# Supplementary material for: Glial activation and inflammation along the Alzheimer’s disease continuum
Source: J Neuroinflammation. 2019 Feb 21;16:46. doi: 10.1186/s12974-019-1399-2 (PMC6383268; doi:10.1186/s12974-019-1399-2)
Supplement: Supplementary file 3 — Figure S2. Between-group comparisons of microglial- and astroglial activation with inflammation based on clinical staging. (PDF 277 kb) [file 12974_2019_1399_MOESM3_ESM.pdf]

Supplementary figure 2: Between-group comparisons of microglial- and astroglial activation with inflammation based on clinical staging

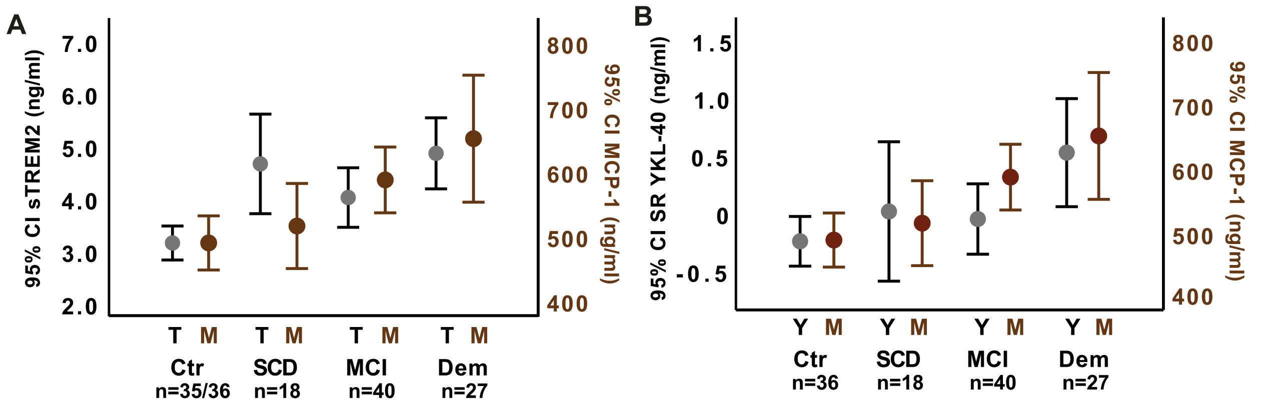

Error bars comparing the microglial (A) and astroglial (B) activation with the pathologic inflammatory response for clinical groups. Early microglial activation without a significant inflammatory response is illustrated by increased sTREM2 (T, black) at the SCD stage, while MCP-1 (M, red) is increased at the MCI stage (A). The astroglial activation marker (YKL-40, Y), however, is only significantly increased at the AD dementia stage (B). The Y-axis with sTREM2 and MCP-1 is reported as CSF concentrations in ng/ml, while the Y-axis for YKL-40 are residuals standardized (SR) for age. Error bars are shown as mean and 95% confidence interval (CI). Abbreviation: Ctr: healthy controls (n=36), SCD: CSF Aβ42+ subjects with subjective cognitive decline (n=19), MCI: CSF Aβ42+ subjects with mild cognitive impairment (n=39), Dem: Aβ42+ subjects with Alzheimer's disease dementia (n=27), T: sTREM2, M: MCP-1, Y: YKL-40.
